# Progress Toward Measles Elimination — Philippines, 1998–2014

**Published:** 2015-04-10

**Authors:** Yoshihiro Takashima, W. William Schluter, Kayla Mae L. Mariano, Sergey Diorditsa, Maricel de Quiroz Castro, Alan C. Ou, Maria Joyce U. Ducusin, Luzviminda C. Garcia, Dulce C. Elfa, Alya Dabbagh, Paul Rota, James L. Goodson

**Affiliations:** 1 Expanded Programme on Immunization, World Health Organization Western Pacific Regional Office, Manila, Philippines; 2 World Health Organization Representative’s Office, Manila, Philippines; 3 National Center for Disease Prevention and Control, Department of Health, Manila, Philippines; 4 Department of Immunization, Vaccines, and Biologicals, World Health Organization, Geneva, Switzerland; 5 Division of Viral Diseases, National Center for Immunization and Respiratory Diseases; 6 Global Immunization Division, Center for Global Health, CDC

In 2005, the Regional Committee for the World Health Organization (WHO) Western Pacific Region (WPR) established a goal to eliminate measles* by 2012 (1). The recommended elimination strategies in WPR include 1) ≥95% 2-dose coverage with measles-containing vaccine (MCV) through routine immunization services and supplementary immunization activities (SIAs)†; 2) high-quality case-based measles surveillance; 3) laboratory surveillance with timely and accurate testing of specimens to confirm or discard suspected cases and detect measles virus genotypes; and 4) measles outbreak preparedness, rapid response, and appropriate case management (2). In the WPR, the Philippines set a national goal in 1998 to eliminate measles by 2008 (3). This report describes progress toward measles elimination in the Philippines during 1998–2014 and challenges remaining to achieve the goal. WHO–United Nations Children’s Fund (UNICEF)–estimated coverage with the routine first dose of MCV (MCV1) increased from 80% in 1998 to 90% in 2013, and coverage with the routine second dose of MCV (MCV2) increased from 10% after nationwide introduction in 2010 to 53% in 2013. After nationwide SIAs in 1998 and 2004, historic lows in the numbers and incidence of reported measles cases occurred in 2006. Despite nationwide SIAs in 2007 and 2011, the number of reported cases and incidence generally increased during 2007–2012, and large measles outbreaks occurred during 2013–2014 that affected infants, young children, older children, and young adults and that were prolonged by delayed and geographically limited outbreak response immunization activities during 2013–2014. For the goal of measles elimination in WPR to be achieved, sustained investments are required in the Philippines to strengthen health systems, implement the recommended elimination strategies, and develop additional strategies to identify and reduce measles susceptibility in specific geographic areas and older age groups.

## Immunization Activities

MCV1 and MCV2 coverage data are reported each year from the 17 regions§ in the Philippines to the National Immunization Programme; national coverage data are reported annually to WHO and UNICEF. WHO and UNICEF use reported data from administrative records and surveys to estimate coverage with MCV1 and MCV2 through routine immunization services. In the Philippines, MCV1 administered at age 9 months was introduced nationwide in 1983, and MCV2 administered at age 12–15 months was introduced nationwide in 2010.¶ WHO-UNICEF–estimated MCV1 coverage increased nationally from 80% in 1998 to 92% during 2004–2008, decreased to 79% in 2011, and increased to 90% in 2013. The number of regions with >95% MCV1 coverage decreased from seven in 2007 to none in 2013. Estimated MCV2 coverage increased nationally from 10% in 2010 to 53% in 2013. During 1998–2014, approximately 76.4 million children received MCV during SIAs. Nationwide SIA coverage was 94%–95% in 1998, 2004, and 2007, but only 84% in 2011 and 91% in 2014. There was significant regional variation in vaccination coverage with MCV1 and with SIAs (Table 1).

## Surveillance Activities

Sentinel site-based surveillance with reporting of line lists of suspected measles cases started in 1989; nationwide measles case-based surveillance with laboratory testing started in 1992, and virus genotyping started in 2010. Key surveillance performance indicators include 1) rate of discarded (i.e., nonmeasles) suspected cases reported per 100,000 population (target: ≥2); 2) percentage of suspected cases with adequate investigation (target: ≥80%); 3) percentage of suspected cases with adequate blood specimens collected for laboratory testing (target: ≥80%); and 4) percentage of suspected cases with results reported within 7 days of the laboratory receiving the specimen (target: ≥80%). During 2009–2011, surveillance performance improved: the discarded non-measles case rate increased from 1.6 to 3.1; the adequate case investigation rate increased from 29.5% to 88.6%; the adequate specimen collection rate increased from 74.1% to 98.0%; and the timeliness of laboratory reporting increased from 53.8% to 72.6%. However, performance declined or varied in 2012 and during the 2013–2014 measles resurgence (Table 1).

## Measles Incidence and Measles Viral Genotypes

**FIGURE 1:**
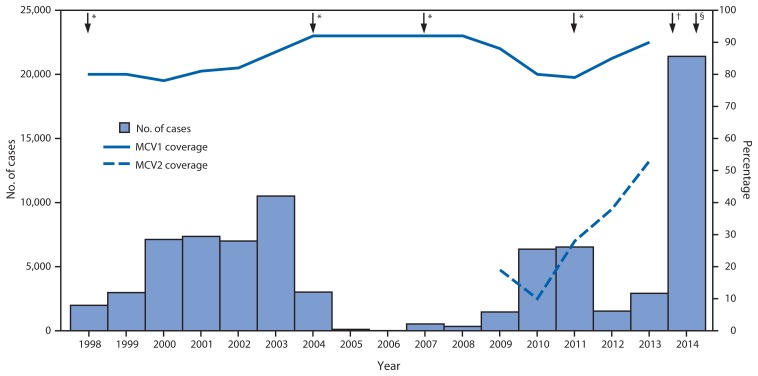
Number of reported measles cases and estimated percentage of MCV1 and MCV2 coverage, by year — Philippines, 1998–2014 **Abbreviation:** MCV = measles-containing vaccine. **Source:** World Health Organization (WHO)–UNICEF estimates of national immunization coverage are available at http://www.who.int/immunization_monitoring/routine/immunization_coverage/en/index4.htm. Estimated coverage with the routine first dose of measles-containing vaccine (MCV1) was among children aged 1 year; estimated coverage with the routine second dose of measles-containing vaccine (MCV2) was among children at the recommended age of administration of MCV2, as per the national immunization schedule. Introduction of MCV2 started in 2009 in Regions 4A, 5, 6, 7, and 12. In 2010, MCV2 was introduced into the routine immunization nationwide; however, reporting was incomplete until the recording/reporting tool was updated in 2012 to accommodate the addition of MCV2. The number of reported measles cases during 1998–2013 is as reported to the World Health Organization (WHO) and UNICEF through the Joint Reporting Form and during 2014 as reported in monthly reports to the WHO Western Pacific Regional Office by December 20, 2014. * Supplementary immunization activities using measles-containing vaccine were implemented in 1998 (nationwide) for children aged 9 months–14 years, 2004 (nationwide) for children aged 9 months–7 years, 2007 (nationwide) for children aged 9–48 months, and using measles-rubella vaccine in 2011 (nationwide) for children aged 9–95 months. ^†^ Outbreak response immunization activities using measles vaccine during January–February 2014 targeting children aged 6–59 months in Calabarzon, Central Luzon, and the National Capital Region. ^§^ Nationwide supplementary immunization activity using measles-rubella vaccine implemented during September 2014 for children aged 9–59 months.

**FIGURE 2:**
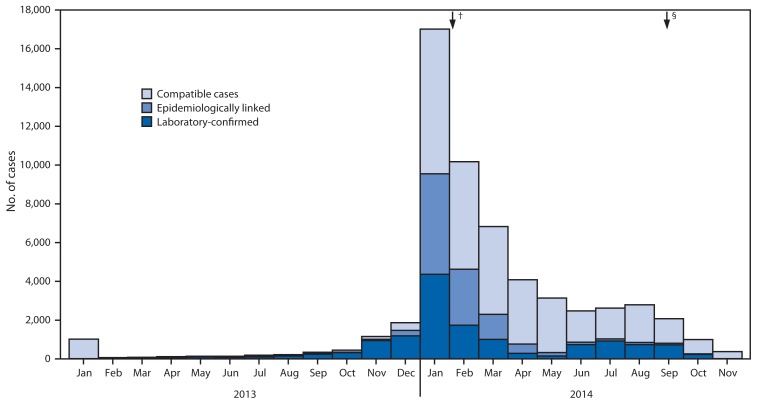
Number* of reported confirmed measles cases, by month of rash onset — Philippines, 2013–2014 **Source:** As reported in monthly reports to the World Health Organization Western Pacific Regional Office by December 20, 2014. * N = 58,389. ^†^ Outbreak response immunization activities using measles vaccine during January–February 2014 targeting children aged 6–59 months in Calabarzon, Central Luzon, and the National Capital Region. ^§^ Nationwide supplementary immunization activity using measles-rubella vaccine implemented during September 2014 for children aged 9–59 months.

During 1998–2014, the number of annual reported measles cases varied in relation to SIAs, declining after SIAs were conducted and then increasing in subsequent years (Figure 1). Overall, annual reported measles cases and incidence per 1 million population decreased from 1,984 and 27.1 in 1998 to nine and 0.1 in 2006 and then increased to 21,403 and 233.2 in 2014. On the basis of SIAs conducted, 2007–2014 can be divided into two periods (Figure 1). During the 2007–2011 inter-SIA period,** 14,142 measles cases were reported. During the 2011–2014 inter-SIA period††, 58,700 measles cases were reported. At the national level, the proportion of measles cases in children aged 9 months–4 years decreased from 38% in the first inter-SIA period to 28% in the second inter-SIA period, and the proportion of measles cases in adolescents and adults aged ≥15 years increased from 18% in the first period to 29% in the second period (Table 2). The nationwide measles resurgence started with outbreaks in Calabarzon (Region 4A), Central Luzon (Region 3), the Cordillera Autonomous Region (CAR), and Western Visayas (Region 6) during the first half of 2013 and spread to many parts of Luzon and Visayas geographical divisions during October–December 2013. Outbreak response immunization activities targeting children aged 6–59 months were implemented in Calabarzon, Central Luzon, and the National Capital Region during January–February 2014; however, by that time the whole country was affected by measles outbreaks (Figure 2). After implementation of the nationwide SIA in September 2014 targeting children aged 9–59 months, 642 (37%) of the 1,719 measles cases during October–December 2014 were in persons aged ≥15 years (Table 2). The predominant measles virus genotype was D3 before 2007, then D9 and G3 during 2007–2009 (4) and D9 during 2010–2012. During 2013–2014, of 69 cases with genotyping, 68 were B3 and one was D9. Genotypes D3 and G3 have not been reported since 2005 and 2010, respectively.

### Discussion

The nationwide measles resurgence in the Philippines during 2013–2014 reflected the insufficient implementation of measles elimination strategies. Persistent low vaccination coverage since 1998 combined with the relatively low level of circulation of measles virus after SIAs resulted in the accumulation of measles-susceptible cohorts of older age children and young adults and a change in the epidemiology of measles in the Philippines. The resurgence highlighted key program challenges: 1) persistent suboptimal MCV1 coverage, 2) low MCV2 coverage since introduction during 2009–2010; 3) suboptimal SIA coverage with large variations in coverage by region; 4) recent SIA target age groups too narrow to interrupt measles virus transmission among older children, evidenced by the proportion of cases occurring outside the SIA target age group; and 5) inadequate outbreak response activities before widespread measles virus transmission started. The failure to achieve high population immunity among the targeted age groups before 2013 contributed to the observed increase in the proportion of measles cases among older children and young adults that indicated a shift in the age of the measles-susceptible population from young children to a wider age group during the nationwide measles resurgence in 2013–2014. This shift will require special strategies for vaccination activities.

In June 2014, the WPR Immunization and Vaccine-Preventable Diseases Technical Advisory Group recommended that countries achieve and maintain ≥95% 2-dose MCV coverage through routine services and periodic SIAs, and, in addition, that endemic countries and countries experiencing nation-wide resurgence 1) update national plans and develop subnational plans with focus on high-risk and measles-susceptible groups; 2) enhance surveillance activities, including rapid case detection and outbreak investigation; 3) annually review and identify districts and age groups with suboptimal population immunity; and 4) increase population immunity by taking corrective actions such as periodic selective immunization activities and more frequent subnational or national SIAs (5). The Technical Advisory Group also recommended maintaining a national outbreak response plan for implementation of timely and prompt response activities.

Based on these recommendations, the Philippines Department of Health proposed new activities for measles elimination in the draft National Immunization Programme Strategic Plan for 2015–2019 (6), with plans to conduct 1) selective immunization activities§§ for children aged 12–35 months in all regions in 2015 and 2) nonselective SIAs for a wide target age group during 2015–2017 in regions with sustained measles virus transmission or identified measles susceptibility among older children and adults. In October 2014, the Department of Health issued an administrative order to strengthen local government capacity to identify measles outbreaks, plan outbreak response activities, and provide health workers with guidance on how to respond appropriately to new outbreaks and sustained measles virus transmission (7). In August 2015, the government will implement a nationwide public school-based measles-rubella-tetanus-diphtheria vaccination of 7th-grade students and establish a school entry immunization check in all public and private schools. Children with incomplete vaccination records at the time of school entry immunization check will be referred to either the school clinic or the nearest health center to receive missed vaccinations.

What is already known on this topic?

In 2005, the World Health Organization (WHO) Regional Committee for the Western Pacific Region (WPR) resolved to eliminate measles by 2012. In the WPR, the Philippines set a national goal in 1998 to eliminate measles by 2008.

What is added by this report?

WHO-UNICEF–estimated coverage with the routine first dose of a measles-containing vaccine (MCV1) increased from 80% in 1998 to 90% in 2013. The estimated coverage with the routine second dose (MCV2) increased from 10% after introduction in 2010 to 53% in 2013. After nationwide supplementary immunization activities (SIAs) in 1998 and 2004, historic lows in numbers and incidence of reported measles cases occurred in 2006. Despite nationwide SIAs in 2007 and 2011, reported cases and incidence generally increased during 2007–2012. During 2013–2014, nationwide measles resurgence occurred, including cases among older children and young adults, because of persistent MCV1 coverage <95%, low MCV2 coverage, and suboptimal MCV coverage in several regions of the country by SIAs conducted during 1998–2011.

What are the implications for public health practice?

Resuming progress toward measles elimination in the Philippines requires sustained investments to strengthen health systems and implement the recommended national and subnational strategies, including achieving and maintaining ≥95% 2-dose MCV coverage, implementing additional strategies for reducing accumulated measles susceptibility among older children and adults, and strengthening surveillance and outbreak response.

The findings in this report are subject to at least two limitations. First, administrative coverage data might be unreliable because of inaccurate estimates of the size of target populations and the reported number of doses delivered. Second, surveillance data underestimate the likely number of cases that occurred because not all persons with measles sought care and were reported through surveillance.

In 2013, the WPR Regional Verification Committee for Measles Elimination¶¶ verified that endemic measles virus transmission had been interrupted for a period of at least 36 months in Australia, Macao [China], Mongolia, and the Republic of Korea. However, during 2013–2014, the measles resurgence in the Philippines led to measles virus importations and increased incidence in several WPR countries including Australia and the Republic of Korea and in countries in other WHO regions*** (8–10). Resuming progress toward regional measles elimination goals requires sustained investments, including strengthening health systems and implementing the recommended strategies in the Philippines.

## Figures and Tables

**TABLE 1:** Coverage with measles-containing vaccine by vaccination delivery strategy and measles surveillance performance — Philippines, 1998–2014

Immunization activities				No. (%) of regions,* by coverage				Range by region (%)	National	
		
Delivery strategy	Vaccine	Target age group	Year	<80%	80%–89%	90%–94%	≥95%		Reported	WUENIC
SIA	M	9 mos–14 yrs	1998	0 (0)	1 (6)	5 (31)	10 (62)	89–105	94	
		9 mos–7 yrs	2004	0 (0)	6 (35)	3 (17)	8 (47)	85–100	95	
		9–48 mos	2007	0 (0)	2 (11)	6 (35)	9 (52)	85–99	95	
	MR	9–95 mos	2011	4 (23)	9 (52)	4 (23)	0 (0)	75–91	84	
	M	6–59 mos	2014†	2 (66)	0 (0)	1 (33)	0 (0)	76–92		
	MR	9–59 mos	2014	0 (0)	2 (11)	9 (52)	6 (35)	82–103	91	
MCV1§	M	9 mos	1998					NA	87	80
			1999					NA	ND	80
			2000					NA	80	78
			2001	12 (80)	3 (20)	0 (0)	0 (0)	49–89	75	81
			2002	11 (68)	5 (31)	0 (0)	0 (0)	59–88	82	82
			2003	8 (50)	6 (37)	2 (12)	0 (0)	66–90	87	87
			2004	6 (37)	9 (56)	1 (6)	0 (0)	75–93	81	92
			2005	1 (5)	5 (29)	7 (41)	4 (23)	78–102	82	92
			2006	0 (0)	7 (41)	7 (41)	3 (17)	82–106	92	92
			2007	1 (5)	4 (23)	5 (29)	7 (41)	72–100	92	92
			2008	0 (0)	8 (47)	5 (29)	4 (23)	81–98	86	92
			2009	1 (5)	11 (64)	5 (29)	0 (0)	63–93	88	88
			2010	5 (29)	8 (47)	2 (11)	2 (11)	73–95	80	80
			2011	5 (29)	8 (47)	4 (23)	0 (0)	70-–94	79	79
			2012	2 (11)	10 (58)	5 (29)	0 (0)	62-–92	85	85
			2013	10 (58)	5 (29)	2 (11)	0 (0)	39–91	90	90
MCV2¶	MMR	12 mos	2010	7 (100)	0 (0)	0 (0)	0 (0)	2–35	10	10
		12–15 mos	2011	16 (100)	0 (0)	0 (0)	0 (0)	5–55	28	28
			2012	17 (100)	0 (0)	0 (0)	0 (0)	11–62	38	38
			2013	17 (100)	0 (0)	0 (0)	0 (0)	5–63	53	53

Surveillance performance			No. (%) of regions, by performance				Range by region	National
	
Performance indicator	Target	Year	0–0.5	0.6–0.9	1–1.9	≥2		
Discarded nonmeasles rate per 100,000 population	≥2	2009	2 (11)	1 (5)	10 (58)	4 (23)	0.1–4.2	1.6
		2010	1 (5)	0 (0)	3 (17)	13 (76)	0.3–21.4	4.3
		2011	1 (5)	1 (5)	5 (29)	10 (58)	0.2–19.1	3.1
		2012	4 (23)	1 (5)	5 (29)	7 (41)	0.1–7.5	2.1
		2013	1 (5)	1 (5)	5 (29)	10 (58)	0.3-–7.6	3.3
		2014	0 (0)	2 (11)	5 (29)	10 (58)	0.8–5.1	3.3
			**<60%**	**60%–69%**	**70%–79%**	**≥80%**		

% suspected cases with adequate investigation**	≥80%	2009	17 (100)	0 (0)	0 (0)	0 (0)	6.3–47.0	29.5
		2010	17 (100)	0 (0)	0 (0)	0 (0)	8.1–59.3	40.6
		2011	8 (47)	4 (23)	3 (17)	2 (11)	23.2–81.6	88.6
		2012	10 (58)	2 (11)	2 (11)	3 (17)	15.7–88.3	57.1
		2013	9 (52)	3 (17)	3 (17)	2 (11)	4.2–81.3	46.1
		2014	12 (70)	1 (5)	3 (17)	1 (5)	5.1–90.4	52.5
% suspected cases with adequate blood specimens††	≥80%	2009	6 (35)	5 (29)	2 (11)	4 (23)	25.3–89.6	74.1
		2010	6 (35)	2 (11)	5 (29)	4 (23)	24.1–95.4	87.1
		2011	4 (23)	1 (5)	6 (35)	6 (35)	27.7–93.9	98.0
		2012	3 (17)	3 (17)	3 (17)	8 (47)	33.9–98.1	80.4
		2013	1 (5)	1 (5)	7 (41)	8 (47)	35.0–95.3	63.2
		2014	5 (29)	3 (17)	4 (23)	5 (29)	18.0–94.7	82.0
% serology laboratory results ≤ 7 days of receipt	≥80%	2009	8 (47)	6 (35)	1 (5)	2 (11)	22.1–100.0	53.8
		2010	15 (88)	1 (5)	1 (5)	0 (0)	27.5–71.9	43.9
		2011	6 (35)	3 (17)	6 (35)	2 (11)	44.7–81.0	72.6
		2012	0 (0)	1 (5)	0 (0)	16 (94)	66.7–100.0	95.3
		2013	0 (0)	1 (5)	4 (23)	12 (70)	68.4–100.0	80.2
		2014	17 (100)	0 (0)	0 (0)	0 (0)	0–23.5	1.3

**Abbreviations:** M = measles vaccine; MCV = measles-containing vaccine; MMR = measles, mumps, and rubella vaccine; MR = measles and rubella vaccine; NA = not available; ND = no data; SIAs = supplementary immunization activities; WUENIC = World Health Organization–UNICEF estimate of national immunization coverage.

* The total number of regions in the Philippines is 17 after 2004.

† SIAs with measles vaccine targeting children aged 6–59 months were carried out only in Regions 3 and 4A and in the National Capital Region.

§ Routine first dose of measles-containing vaccine. MCV1 coverage by region is not available before 2001.

¶ Routine second dose of measles-containing vaccine. Introduction of MCV2 started in 2009 in Regions 4A, 5, 6, 7, and 12. In 2010, MCV2 was introduced into the routine immunization nationwide; however, reporting was incomplete until the recording/reporting tool was updated in 2012 to accommodate the addition of MCV2.

** Adequate investigation is defined as investigation initiated within 48 hours of notification, with collection of all 10 core variables (case identification, date of birth/age, sex, place of residence, vaccination status or date of last vaccination, date of rash onset, date of notification, date of investigation, date of blood specimen collection, and place of infection or travel history).

†† Adequate specimens are minimum of 5 ml of blood sample for older children and adults and 1 ml for infants and younger children or dried blood sample with at least three fully filled circles on filter paper collected within 28 days of rash onset.

**TABLE 2:** Reported measles cases* before and after supplementary immunization activities (SIAs)† in 2011 and 2014, by age group — Philippines, November 1, 2007–December 31, 2014

Age group	Time Period					

	Nov 2007–May 2011		June 2011–Sept 2014		Oct 2014–Dec 2014	
		
	No.	(%)	No.	(%)	No.	(%)
0–8 mos	1,831	(13)	18,033	(31)	462	(27)
9 mos–4 yrs	5,412	(38)	16,671	(28)	357	(21)
5–9 yrs	2,664	(19)	2,846	(5)	115	(7)
10–14 yrs	1,222	(9)	4,188	(7)	141	(8)
15–29 yrs	2,073	(15)	12,552	(21)	450	(26)
30–39 yrs	331	(2)	3,866	(7)	169	(10)
≥40 yrs	102	(1)	482	(1)	23	(1)
No data	507	(4)	62	(0)	2	(0)
**Total**	**14,142**	**(100)**	**58,700**	**(100)**	**1,719**	**(100)**

* Includes reported measles cases that were laboratory confirmed, epidemiologically linked, and either clinically confirmed (2007–2012) or clinically compatible (2013–2014). Both clinically confirmed and clinically compatible cases were suspected cases with fever and maculopapular (nonvesicular) rash and one of cough, coryza, or conjunctivitis for which no adequate clinical specimens were taken and that were not linked epidemiologically to laboratory-confirmed cases of measles.

† SIAs were implemented in October 2007 (nationwide) targeting children aged 9–48 months, during April–May 2011 (nationwide) for children aged 9–95 months, during January–February 2014 (in Regions 3 and 4A and in the National Capital Region) for children aged 6–59 months, and in September 2014 (nationwide) for children aged 9–59 months.

## Footnotes

* Measles elimination is defined as the absence of endemic measles virus transmission in a defined geographical area (e.g. region or country) for ≥12 months in the presence of a well-performing surveillance system..

† Measles SIAs generally are carried out using two target age ranges. An initial, nationwide catch-up SIA targets all children aged 9 months–14 years, with the goal of eliminating susceptibility to measles in the general population. Periodic follow-up SIAs then target all children born since the most recent SIA. Follow-up SIAs generally are conducted nationwide every 2–4 years and target children aged 9–59 months; their goal is to eliminate any measles susceptibility that has developed in recent birth cohorts and to protect children who did not respond to the first measles vaccination.

§ The 17 administrative regions in the Philippines include the Cordillera Autonomous Region (CAR), the National Capital Region (NCR), Region 1 (Ilocos), Region 2 (Cagayan Valley), Region 3 (Central Luzon), Region 4A (Calabarzon), Region 4B (Mimaropa) and Region 5 (Bicol) in Luzon; Region 6 (Western Visayas), Region 7 (Central Visayas), and Region 8 (Eastern Visayas) in Visayas; and Region 9 (Zamboanga Peninsula), Region 10 (Northern Mindanao), Region 11 (Davao), Region 12 (SOCCSKSARGEN), Caraga, and ARMM (Autonomous Region in Muslim Mindanao) in Mindanao.

¶ MCV2 was introduced in Regions 4A, 5, 6, 7, and 12 in 2009 and introduced nationwide in 2010.

** From the end of the nationwide SIA targeting children aged 9–48 months in October 2007 until the completion of the nationwide SIA targeting children aged 9–95 months in April–May 2011.

†† From the end of the nationwide SIA in April–May 2011 until the completion of the nationwide SIA targeting children aged 9–59 months in September 2014.

§§ Selective immunization activities will be carried out for children aged 12–35 months who have not yet been fully vaccinated with 2 doses of measles-containing vaccines while nonselective SIAs will be done for any person in the target age group regardless of past vaccination history.

¶¶ In 2005, the Regional Committee for the WHO WPR established a goal for measles elimination by 2012, and a Regional Verification Committee (RVC) was established in 2013. In March 2015, the RVC verified that endemic measles virus transmission had been interrupted for a period of at least 36 months in Brunei Darussalam, Cambodia, and Japan.

*** During 2013–2014, a total of 17 countries in four WHO regions reported measles virus genotype B3 in persons who had a history of recent travel to the Philippines.
